# Barriers and Facilitators to Maintaining Physical Activity After an mHealth Intervention for People Poststroke or Transient Ischemic Attack: Reflexive Thematic Analysis

**DOI:** 10.2196/87493

**Published:** 2026-05-27

**Authors:** Hanna Lagerlund, David Moulaee Conradsson, Charlotte K Häger, Lydia Kwak, Lisa Holmlund

**Affiliations:** 1 Division of Physiotherapy, Department of Neurobiology, Care Sciences and Society, Karolinska Institutet, Alfred Nobels Allé 23, 23100, Stockholm, SE-141 52, Sweden, 46 852486614; 2 Medical Unit Allied Health Professionals, Women’s Health and Allied Health Professionals Theme, Karolinska University Hospital, Stockholm, Sweden; 3 Department of Community Medicine and Rehabilitation, Umeå University, Umeå, Västerbotten, Sweden; 4 Department of Diagnostics and Intervention, Umeå University, Umeå, Västerbotten, Sweden; 5 Unit of Intervention and Implementation Research for Worker Health, Institute of Environmental Medicine, Karolinska Institutet, Stockholm, Sweden; 6 Division of Occupational Therapy, Department of Neurobiology, Care Sciences and Society, Karolinska Institutet, Stockholm, Sweden

**Keywords:** digital interventions, digital health, mobile health, mobile apps, photo-elicitation, physical activity maintenance, stroke

## Abstract

**Background:**

Regular physical activity is critical for preventing secondary stroke following a stroke or transient ischemic attack (TIA). Although mobile health (mHealth) interventions have shown promise for promoting short-term increases in physical activity, evidence on their long-term effects and the mechanisms that support sustained behavior change remains limited. In particular, little is known about how people poststroke or TIA integrate the skills, knowledge, and habits gained through mHealth interventions into their daily lives once structured intervention support ends.

**Objective:**

This study aimed to explore the perceived barriers and facilitators of maintaining physical activity among individuals poststroke or TIA after completing an mHealth intervention.

**Methods:**

A qualitative approach was used, involving a strategic sample of 12 participants recruited after they had completed a 6-month mHealth intervention for people poststroke or TIA. The intervention included supervised physical therapy with mHealth support for physical exercises and behavior change (eg, counseling, goal-setting, and self-monitoring), followed by a 6-month postintervention period with access to self-managed mHealth support. To enable richness and depth in participants’ accounts, the dataset consisted of 2 semistructured interviews conducted 3 months and 6 months after completing the intervention, along with participant-generated photographs. Between the interviews, participants took photos to reflect their experiences of maintaining physical activity after the intervention. These images served as prompts for dialog and reflection during the second interview. Data were analyzed using reflexive thematic analysis.

**Results:**

We generated 3 themes: building experience and knowledge to maintain physical activity, staying physically active in a complex life situation, and the meaning of context for maintaining physical activity after the intervention. Barriers and facilitators were conceptually integrated into a comprehensive understanding of maintaining physical activity, symbolized by the metaphor of a soap bubble, which requires persistence and a supportive environment to stay afloat. The complexity of participants’ health and life situations created barriers to maintaining physical activity. To overcome these barriers, developing personal strategies within a supportive social and physical context was crucial. This development was facilitated by the mHealth intervention, which enabled knowledge acquisition of the principles of physical activity after stroke or TIA, along with increased awareness of its health benefits.

**Conclusions:**

mHealth interventions for people poststroke and TIA can serve as a catalyst for physical activity engagement and an enhancer of the knowledge and experience necessary to maintain physical activity after the intervention. Despite health-related and contextual barriers, participants may use personalized strategies, supported by their social and environmental context, to navigate these challenges. These insights highlight opportunities for future mHealth interventions to strengthen the interaction between individuals and their environment and empower tailored strategies for behavior change and long-term physical activity maintenance.

## Introduction

Stroke is the third leading cause of death and disability globally, representing a major public health challenge that demands effective preventive strategies [1]. As a modifiable risk factor for recurrent stroke and transient ischemic attack (TIA), physical activity plays a vital role in secondary stroke prevention [2]. However, many individuals poststroke and TIA fail to meet recommended physical activity levels [3,4]. This highlights the need for interventions that not only promote physical activity but also support its long-term maintenance [5,6].

Physical activity refers to all movements produced by skeletal muscles that require energy expenditure, including leisure activities, commuting, and household chores [7,8]. In contrast, physical exercise is a subcategory of physical activity that is structured, goal-directed, and aimed at enhancing physical fitness [7,8]. In the context of digital transformation of care, mobile health (mHealth) has emerged as a promising approach to promoting physical activity among people poststroke or TIA [9]. There is recent evidence that mHealth interventions can support people poststroke or TIA to establish exercise routines and increase physical activity in the short term [10,11], whereas strategies to sustain these improvements over time remain underexplored [5]. Furthermore, most mHealth trials rely on self-management alone [11], which contrasts with the expressed need for therapeutic support from professionals experienced in stroke rehabilitation, as reported by people poststroke or TIA [12]. In line with this, a recent overview showed that multicomponent interventions combining therapist-led sessions with self-management provide moderate evidence for improving physical activity and reducing cardiac events poststroke or TIA [13]. Therefore, to develop mHealth interventions that effectively support long-term physical activity after stroke or TIA, it is crucial to deepen our understanding of how people poststroke or TIA experience the maintenance of physical activity through self-management after completing an intervention that included therapeutic support. Moreover, more knowledge is needed about how people poststroke or TIA integrate the skills, knowledge, and habits acquired through mHealth interventions into their daily lives once structured support ends.

Physical activity maintenance could be operationalized using behavioral indicators such as meeting predefined thresholds (eg, 150 minutes of physical activity per week), consistency over a specified observation period, and regularity of engagement [14]. Several factors are known to influence physical activity engagement among individuals poststroke and TIA. Facilitators include peer support and self-efficacy for participation in physical activity, while barriers include stroke-related impairments and inadequate public transportation to physical activity venues [15,16]. Maintaining physical activity over time may involve distinct factors to those influencing the initiation of physical activity [17]. Developing a self-identity as a physically active person and incorporating flexibility into routines have, for example, been identified as facilitators specific to long-term physical activity maintenance in adults [17]. Moreover, among older adults, scheduled physical activity, encouragement from spouses, and access to both indoor and outdoor environments have been shown to facilitate the maintenance of physical activity, while the absence of these factors acted as barriers [18]. These findings of person-environment interaction in maintaining physical activity are in line with the social cognitive theory (SCT), which posits that environmental support and individual self-regulation are important for behavior change [19]. SCT also emphasizes how personal factors, a person’s behavior (what they do or think), and the environment in which they act interact reciprocally, meaning that changes in one of these factors influence the others [19]. To the best of our knowledge, there is currently no research that specifically addresses the maintenance of physical activity following an mHealth intervention in individuals poststroke or TIA.

This study builds on a telehealth intervention from Australia, *i-REBOUND – Let’s Get Moving!*, designed to support the promotion and maintenance of physical activity in people poststroke or TIA through supervised exercise and support for self-management through behavior change techniques [20]. In prior work by our group, i-REBOUND was adapted into a digital intervention combining physical therapist–led sessions (supervised exercise and counseling) and self-managed mHealth support (eg, activity diary, prerecorded exercise videos), delivered via a mobile phone app [12]. The mHealth version of the i-REBOUND intervention was found to be feasible, acceptable, and successful at delivering the intervention in an mHealth format [21,22]. To further refine the mHealth version of the i-REBOUND intervention and address the knowledge gap regarding the maintenance of physical activity following mHealth interventions, it is important to understand which factors positively and negatively influence maintained physical activity. This study aimed to explore the perceived barriers and facilitators to maintaining physical activity among individuals poststroke or TIA after completing the mHealth version of the i-REBOUND intervention.

## Methods

### Study Design

This study is grounded in a constructivist epistemology, in which knowledge is understood as coconstructed through social interaction between participants and researchers rather than discovered as an objective truth. The dataset was generated through repeated semistructured interviews and participant-generated photos [23], and a reflexive thematic approach was applied in the data analysis [24,25].

The authors form an interdisciplinary team of 4 women and 1 man, all based in academic settings at Swedish universities. Four of the authors also have experience in clinical practice within physiotherapy (CKH, DMC, and HL) or occupational therapy (LH). All are experienced in intervention and implementation research, including mHealth (CKH and DMC) and knowledge of behavior change theories (HL and LK). In line with constructivism and reflexive thematic analysis [26], themes are viewed as actively generated through an interpretative and reflexive analytic process that draws on participants’ accounts and researchers’ positionality. Participants with experience of the mHealth version of the i-REBOUND intervention were integral to the knowledge production process, and we acknowledged our active role as researchers in shaping the analysis. The study’s reporting follows the Reflexive Thematic Analysis Reporting Guidelines [27].

### Participants

We strategically recruited 12 participants (10 women, 2 men) from the experimental group (n=57) of a feasibility randomized controlled trial (n=114) after participating in the mHealth version of the i-REBOUND intervention for people poststroke and TIA [21]. Inclusion criteria for the feasibility randomized controlled trial were adults who (1) experienced a stroke or TIA between 3 months and 10 years prior to the study, (2) lived at home, (3) had access to a reliable internet connection, (4) could walk short distances indoors with or without a walking device, (5) could use a smartphone, and (6) could self-identify through a Swedish secure self-authentication app [28]. Exclusion criteria were (1) currently meeting recommended physical activity levels (ie, at least 150 minutes per week of moderate physical activity or at least 75 minutes per week of vigorous intensity physical activity [29]) or had (2) severe health conditions and/or (3) significant cognitive impairments [28]. Physical activity levels of the participants were assessed via a telephone interview, and individuals were eligible for inclusion if they self-reported not meeting the recommended 150 minutes of moderate-intensity physical activity per week based on frequency, intensity, and duration. The strategic sampling aimed to achieve diversity in age, sex, physical activity level, geography, and stroke or TIA diagnosis. Twelve participants were identified and agreed to participate. Among the recruited participants, 10 regions across Sweden were represented, and the participants were aged 56 years through 80 years at the start of the intervention. Participant characteristics are presented in Table 1.

**Table 1.** Participant characteristics at baseline (n=12), recruited from the experimental group of a feasibility randomized controlled trial.

Characteristics	Value
Age^a^ (years), mean (min-max)	70 (56‐80)
Women, n (%)	10 (83)
Clinical diagnosis, n (%)	
Stroke	8 (67)
TIA^b^	4 (33)
Duration since stroke/TIA diagnosis (years), mean (min-max)	4 (0.67‐9.67)
Having a comorbidity^c^ (yes), n (%)	11 (92)
Highest level of education, n (%)	
Primary school	1 (8)
Secondary school	6 (50)
University	5 (42)
Employed (yes), n (%)	4 (33)
Living with someone (yes), n (%)	8 (67)
Living in a rural area (yes), n (%)	3 (25)
Modified Rankin Scale^d^ (0‐6), mean (min-max)	1 (0‐1)
Fatigue Severity Scale^e^ (1-7), mean (min-max)	3.7 (1.8‐5.4)
Previous experience with digital health care services, n (%)	7 (58)
Daily use of internet and mobile apps, n (%)	8 (67)
Steps per day, mean (min-max)	6901 (1048‐13,897)

a All demographic data were collected at baseline, between September and November 2022, prior to the start of the mobile health version of the i-REBOUND intervention.

b TIA: transient ischemic attack.

c Comorbidity is defined as the co-existence of one or more conditions alongside the primary condition (stroke/TIA) [30].

d Assessment of the level of stroke disability according to the Modified Rankin Scale [31].

e Fatigue was assessed using the 9-item version of Fatigue Severity Scale [32].

### The mHealth Version of the i-REBOUND Intervention

For full intervention details, see the study protocol [28]. The mHealth version of the i-REBOUND intervention aimed to support home-based exercise and physical activity for individuals poststroke or TIA. The intervention was delivered through an mHealth app called the STAAR (Stroke Treatment through Active and Accessible Rehabilitation) app. During the 6-month intervention, participants received digital supervised support from a physical therapist and had access to self-managed mHealth support. The participants attended weekly exercise sessions of moderate to vigorous intensity (months 1 to 3: two 25-minutes sessions per week; months 4 to 6: one 25-minute session per week) and monthly individual 30-minute counseling sessions led by a physical therapist. The counseling sessions focused on setting physical activity goals, discussing progression, and developing self-management strategies to support maintained physical activity engagement. Examples of goals were “Gym training twice a week on Mondays and Wednesdays. 20 minutes of aerobic exercise and 40 minutes of strength training. Borg scale 13‐14.” and “Brisk walks two to three times per week, 30 to 60 minutes per session.*”* To further support the promotion of physical activity, the app provided self-managed mHealth support, including educational videos, an activity diary for self-monitoring, and prerecorded exercise videos. During the intervention, a chat function enabled contact with the physical therapist. An action plan, including long-term goals, for maintaining engagement in physical activity postintervention was formulated with support of the physical therapist at the final counseling session. After the intervention, participants continued to have access to self-managed mHealth support, although no further contact with the physical therapist was provided.

### Dataset Generation

Semistructured interviews were conducted in Swedish at 3 months and 6 months postintervention. Interviews were carried out via digital video calls by the first author, a PhD candidate and experienced geriatric physical therapist, between June 2023 and November 2023. In the 6-month interview, participant-generated photo elicitation was used [23]. The first author had brief contact with the participants prior to the interviews, due to her involvement in the data collection in the feasibility randomized controlled trial.

The first and last authors developed and pilot-tested the interview guides. The 3-month interview covered the following conceptual areas: (1) the participants’ perceptions of maintaining physical activity after the intervention, (2) barriers and facilitators to maintaining physical activity after the intervention, and (3) expectations and concerns about maintaining physical activity after the intervention. Examples of interview questions were “What has been your experience of being physically active after the intervention ended?” and “Can you tell me what facilitated regular physical activity for you after the intervention ended?” Before the 6-month interview, the participants were encouraged to take 3 to 5 photos reflecting how they perceived maintaining their physical activity after completing the mHealth version of the i-REBOUND intervention. After a brief reflection on their current physical activity level, the participants were asked to reflect on one photo at a time. Open-ended questions were inspired by Green et al [33] and included “Tell me about this photo—why did you choose to bring this photo?” “Tell me what could prevent you from doing this activity regularly?” and “Was there anything missing in the intervention to make this activity regular?” To enhance the richness of the descriptions, participants were prompted with follow-up questions and encouraged to elaborate on their responses using examples of situations. The interviews lasted between 40 minutes and 90 minutes and were audio-recorded. Field notes were written after each interview. Interviews were transcribed and anonymized before the data analysis.

### Data Analysis

Analysis followed reflexive thematic analysis, based on the 6-phase process outlined by Braun and Clark [24,25]. The primary focus was on the transcribed text; however, photos were analyzed alongside participants’ interviews to add context and generate a richer understanding of the participants’ verbal accounts [34]. The software program N-Vivo 15 was used to organize the transcribed data. In phase one, *familiarization with data*, the first author familiarized herself with the dataset by carefully reading and listening to all interviews while simultaneously taking field notes. In this phase, discussions were continuously held with the last author, who read a sample of the interviews. In phase two, *generating initial codes,* the first author, in collaboration with the last author, identified and labeled parts of the text that captured meaningful experiences related to the research aim. The third phase, *generating initial themes,* included reflexive discussions between the first and the last authors in which the initial codes were reviewed and refined through merging, renaming, and grouping codes to explore patterns of meaning across the dataset. Phases four and five, *theme development and review* and *theme refinement,* were carried out through a continuous iterative process with ongoing discussions among all authors, involving data, codes, and themes. The focus was on identifying clear patterns within each theme, ensuring conceptual consistency and coherence, and naming the themes [26]. In these later phases, photos were used to provide an overall interpretation of physical activity maintenance and to develop themes. For example, a reflexive discussion around a photo of a soap bubble and the story aligned with it helped capture an overall understanding of the complexity of maintaining physical activity and the environmental influence on this process. Further, there were many photos illustrating aspects of the environment. Through reflexive discussions about what these photos and stories meant for staying physically active after an mHealth intervention, we could further develop themes. In analyzing person-environment interactions, an existing behavior change theory, SCT [19], was used to inform data interpretation and further develop themes in line with reflexive thematic analysis [26,27]. For example, photos and stories about social and physical environments were useful for understanding aspects such as reinforcement and the role of social support in the first and third themes. In this way, the photos not only contextualized participants’ narratives but also contributed analytically by expanding the understanding of barriers and facilitators to maintaining physical activity after the intervention. In phase six, *writing up*, the final report was produced, and all authors read, discussed, and contributed to the writing through several rounds of the manuscript.

### Ethical Considerations

Ethical approval for the study was obtained from the Swedish Ethical Review Authority (dnr 2020‐05062 and 2022-07270-02). The research followed the Declaration of Helsinki, and careful consideration was given to ensure the integrity and well-being of the participants throughout the research process. Prior to their consent, all participants were provided with verbal and written information about the study, including their right to withdraw their participation at any time. No compensation for participation was provided. After transcription, the data were de-identified by removing identifiable information from the transcripts, and photographs were selected to ensure participants’ confidentiality.

## Results

### Overview

**Figure 1.**
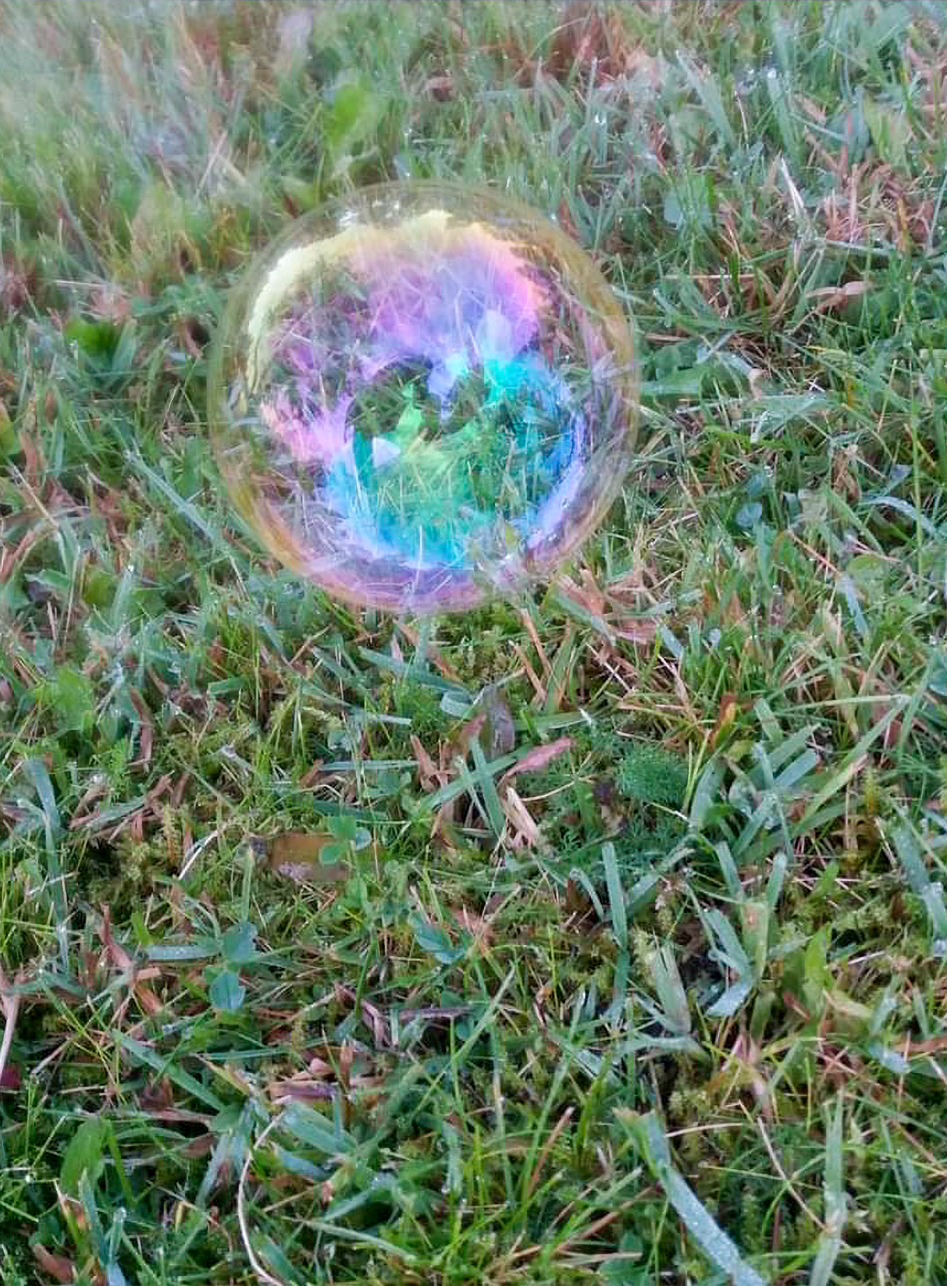
A soap bubble illustrating the inevitable fragility of maintaining physical activity (Female participant, 67 years old, transient ischemic attack).

The overall understanding of maintaining physical activity is symbolized by a photo of a soap bubble, first illustrated by a participant emphasizing the inevitable fragility of staying physically active (Figure 1): “For me this soap bubble is the a...almost impossible, a soap bubble doesn’t last forever, I mean it really doesn’t. But for how long can you hold on, for how long can you actually hold on*”* (female participant, 67 years old, TIA). Intervention mechanisms and personal factors contributed to the persistence of the soap film. At the same time, contextual conditions created an environment that kept the bubble afloat, even in the face of barriers in participants’ life situations. We generated 3 themes through analysis, illustrating the barriers and facilitators to maintaining physical activity after completing the i-REBOUND intervention (Table 2).

**Table 2.** Themes, subthemes, and barriers and facilitators to maintaining physical activity after completing the mobile health (mHealth) version of the i-REBOUND intervention.

Themes and subthemes	Barriers	Facilitators
Building experience and knowledge to maintain physical activity	Limited experience with physical exercise	Regular experience with physical exercise over the 6-month intervention Positive experience with the health benefits of physical activity Knowledge acquisition of principles of physical exercise after stroke and TIA^a^ Knowledge acquisition on how to use daily routines and activities as a resource of physical activity Previous experiences with physical exercise after stroke or TIA
Staying physically active in a complex life situation		
Integrating physical activity into daily life amid health challenges	Health impact of poststroke impairments and comorbidity Impact of engagements, roles, and responsibilities in daily life Lack of personal strategies for maintaining physical activity	—^b^
Strategies for integrating physical activity into daily life	Minimal added value of the self-managed support in the STAAR^c^ app	Belief in one’s capability to be physically active Management of poststroke impairments and comorbidities Self-monitoring through hand-written exercise logs or the step counter on the phone Adhering to a structured yet feasible action plan
The meaning of context for maintaining physical activity after the intervention	Discontinued support from a physical therapist Weather and seasonal changes	Involvement of significant others in the social network as physical activity companions A supportive social context encouraging engagement in physical activity A designated place for performing physical activity

a TIA: transient ischemic attack.

b In this subtheme, only barriers were described.

c STAAR: Stroke Treatment through Active and Accessible Rehabilitation.

### Building Experience and Knowledge to Maintain Physical Activity

A persistent soap film served as a metaphor for gaining experience and acquiring knowledge through participation in the intervention. The continuous interaction with the physical therapist during individual counseling and regular exercise sessions over 6 months provided opportunities for habit formation. The regular engagement in exercise also enabled the participants to experience the benefits of physical activity for health and managing daily life. Along with a deeper understanding of the principles of physical exercise, the intervention components and mechanisms of change supported a stable foundation to maintain regular physical activity after the intervention.

**Figure 2.**
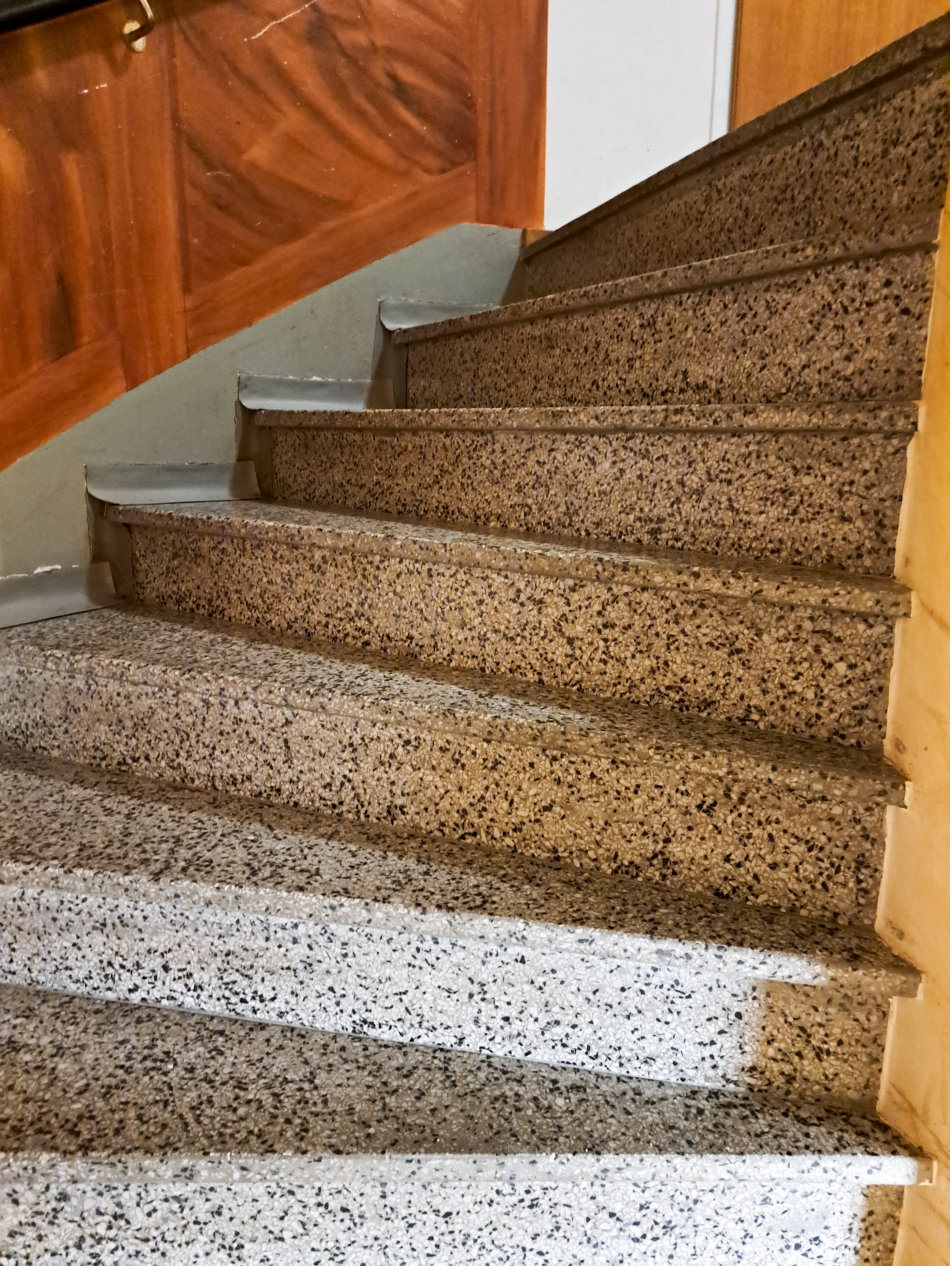
A staircase reinforcing physical activity maintenance for sustained ability to climb stairs (female participant, 66 years old, stroke).

The regular physical exercise sessions at higher intensity levels had served as a catalyst for physical activity and reinforced habit formation for continued activity after the intervention. In addition, being regularly active over time enabled the participants to experience and recognize the health benefits of physical activity. Examples of such benefits included reduced pain levels, increased energy for daily activities, and an overall feeling of improved well-being. The catalyst for change through these experiences was felt to be beneficial regardless of previous engagement in physical activity. One participant shared how the intervention was an awakening of the benefits of being regularly active*:* “I mean, I did move around before as well, but this has still given me a bit of…a little step forward, in some way. I think so. Uh, a wake-up call. That this is…this is good. It’s good for me to do these things” (female participant, 76 years old, stroke). In line with experiencing health benefits, the participants also became aware of the importance of increased strength for managing daily activities. This awareness was reflected in a photo of stairs (Figure 2). The participant lived in a building without an elevator, and the photo symbolized how regular physical activity was a prerequisite to sustaining the ability to climb stairs and accessing the apartment. This example illustrated how maintaining physical activity was perceived not only as beneficial for health but also as a means for sustaining important abilities for their daily life.

**Figure 3.**
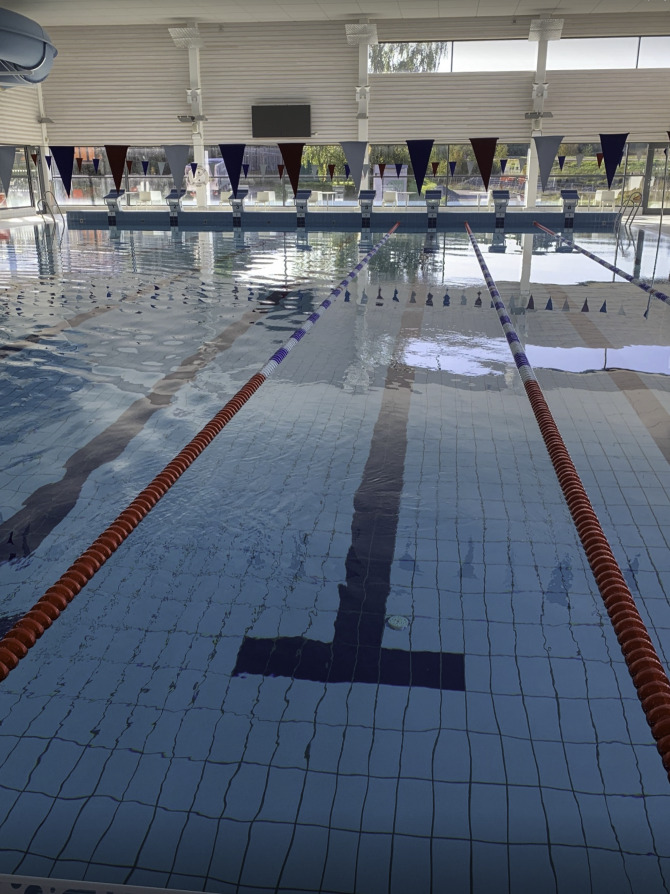
A swimming pool illustrating a modified exercise routine for increased exercise intensity (female participant, 76 years old, stroke).

During the intervention, the participants developed an understanding of the importance of exercise intensity and the benefits of having an increased heart rate during physical exercise. Participants used this knowledge to adapt their physical exercise routines to meet recommendations for physical activity. Moreover, participants learned how to use daily activities as opportunities for physical activity. Walking, in particular, was an important and easily accessible activity that could be adapted, for example, by replacing slow-paced dog walks with brisk power walks without the dog. Another example of modifying an existing routine, illustrated by a photo of a swimming pool (Figure 3), was the incorporation of faster swimming intervals to enhance intensity. In addition, the introduction of tools, such as the Borg scale [35], and specific exercises provided a foundation for a personalized exercise routine and set the stage for progression. The familiarity and simplicity of the intervention’s exercises supported maintenance, as they were easy to remember and carry out, and the Borg scale was a practical tool for the participants to monitor and achieve their target intensity levels.

The participants entered the intervention with different levels of prior knowledge for their knowledge acquisition. Participants with previous exercise experience could continue to develop knowledge, and the intervention’s supervised support reinforced their understanding. In addition, the supervised sessions helped these participants develop realistic expectations for physical exercise, adapted to their age and current physical ability. One participant reflected: “This thing about setting the training at a, yeah…a level that’s just right. That made it possible to do things differently. It just felt…I mean, mentally it was different*”* (male participant, 70 years old, TIA). As the quote illustrates, the knowledge acquired during the intervention prompted a shift in mindset that facilitated acceptance of exercise at a more manageable level. For the participants with limited experience of physical exercise, the intervention served as an introduction to establishing exercise routines and contributed to the acquisition of knowledge on exercise and physical activity, which supported their ability to maintain physical activity after the intervention.

### Staying Physically Active in a Complex Life Situation

#### Overview

Maintaining physical activity after the intervention could be challenging due to participants’ health and contextual circumstances. The participants’ life situations often involved poststroke impairments, comorbid conditions, and daily roles and responsibilities that continuously competed with time and energy for physical activity. Facilitators to overcome these barriers, some of them introduced by the intervention, included a variety of personal strategies to support maintained physical activity. These barriers and facilitators are illustrated in two subthemes: *Integrating physical activity into daily life amid health challenges* and *Strategies for integrating physical activity into daily life*.

#### Integrating Physical Activity Into Daily Life Amid Health Challenges

Health challenges posed considerable barriers to maintaining physical activity after the intervention. Comorbidities, such as heart disease, arthritis, and cancer, meant a balancing act between how the consequences of their condition affected physical ability and the struggle to maintain being physically active. One participant described how multiple hospital visits, medical treatment, and an exercise ban prescribed by the physician disrupted all plans for exercising: “Because of all that stuff—I was about to say—with atrial fibrillation and all that…and the inflammation I had, which threw off…or ruined all that with the training. It [medical problems] has continued…” (male participant, 70 years old, TIA). In addition to disrupting physical activity, health challenges required the participants to engage in physical activity at a lower level than originally intended, according to the goals set at the end of the intervention. Fatigue and short-term illness, such as colds, infections, and musculoskeletal pain, forced the participants to rebuild their physical activity routines repeatedly, as one participant said: “I’ve been interrupted all the time, that’s the thing. As soon as I start and get going a little then [laughs]…something happens” (female participant, 71 years old, stroke). These constant interruptions both affected the ability to maintain physical activity and, at times, undermined the motivation to continue the efforts.

**Figure 4.**
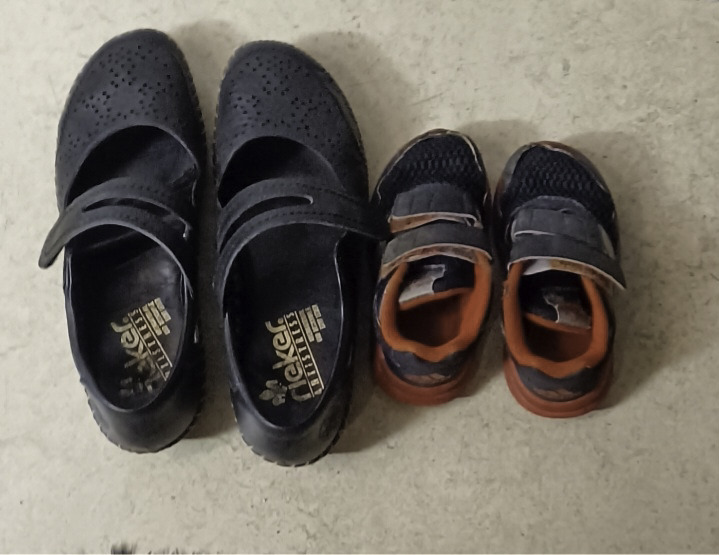
Shoes side by side illustrating engagement with grandchildren as a facilitator of physical activity (female participant, 67 years old, transient ischemic attack).

**Figure 5.**
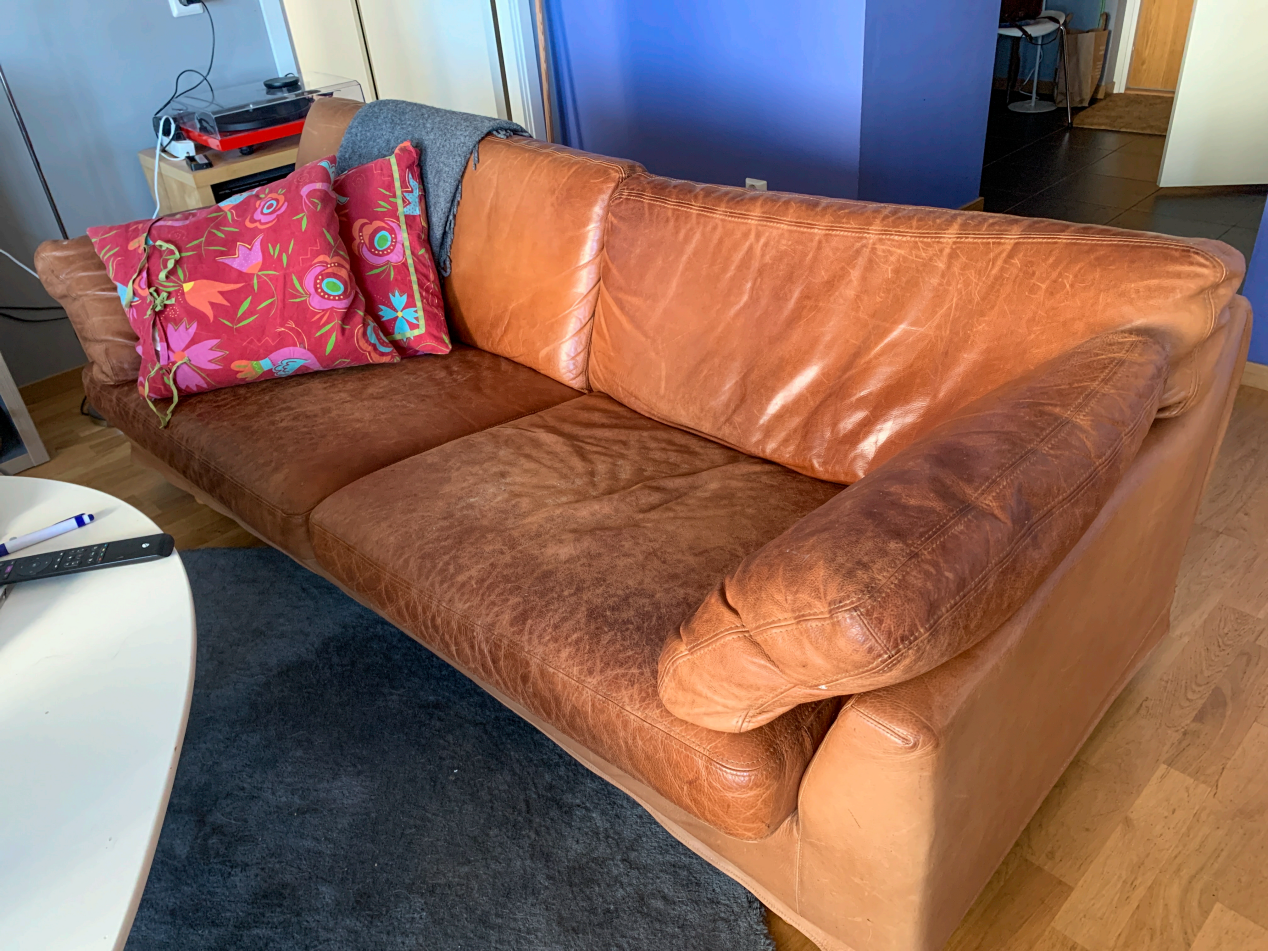
A living room sofa symbolizing sedentary routines competing with physical activity engagement (female participant, 80 years old, transient ischemic attack).

Maintaining physical activity was also influenced by a pull of other engagements, roles, and responsibilities of daily life, which could function as both barriers and facilitators for physical activity. On the one hand, engaging in activities such as gardening and caring for grandchildren facilitated physical activity. One participant shared a photo of 2 pairs of shoes placed next to each other (Figure 4), illustrating how activities with her grandchildren, such as going for a bike ride with them instead of driving, facilitated physical activity. However, for maintaining intensive exercise, it was necessary to integrate such exercise sessions into daily life at large. On the other hand, participants also reflected that the pull of sedentary daily activities was a barrier, sometimes stronger than the push to engage in regular physical activity. This insight was coupled with a sense of failure and personal responsibility for not meeting the expectations after the intervention. One participant exemplified this struggle with a photo of her sofa (Figure 5):

*I don’t mind engaging in physical exercise, but it is…It’s so hard to make it happen at home, I don’t know why, but I’m kind of generally lazy and sluggish, I have to say, so it ends up with me sitting on the couch watching TV instead. Unfortunately*.

[Female participant, 80 years old, TIA]

**Figure 6.**
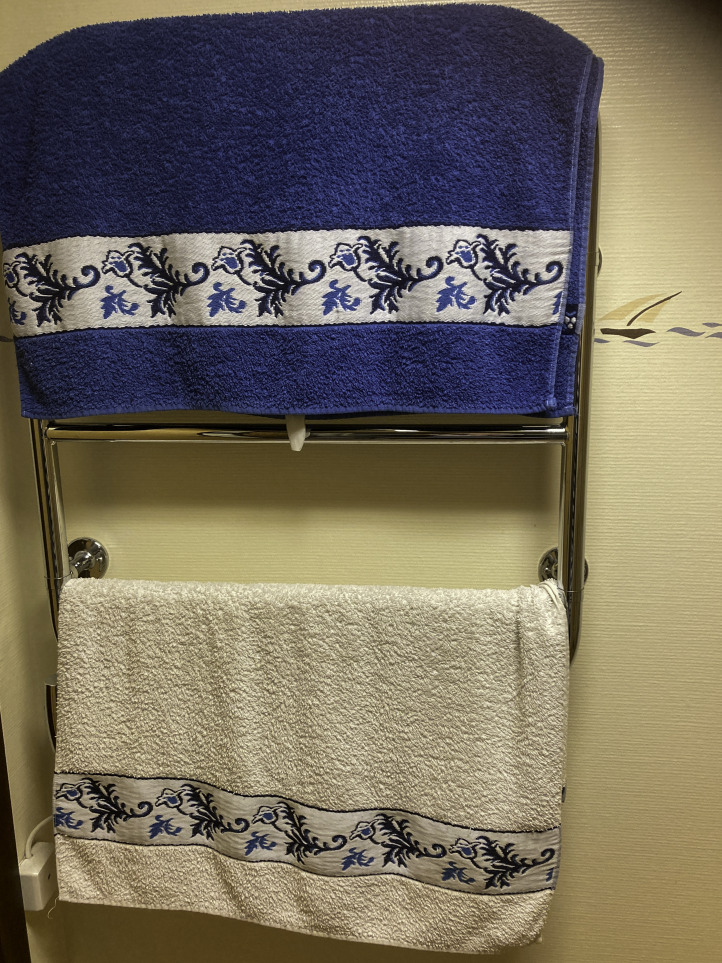
Two towels on a towel holder symbolizing the struggle to allocate time for physical exercise (female participant, 76 years old, transient ischemic attack).

Similarly, the participants’ roles and responsibilities could pose barriers for maintaining regular physical activity; for example, one woman with the responsibility of caring for a sick spouse said: “Yeah, … taking care of the home. Mhm. I have a sick husband, so…Everything kind of falls on me, in a way*”* (female participant, 76 years old, stroke). The responsibilities for household chores meant that the routines established during the intervention were put on hold for her, and given her husband’s condition, leaving the house was difficult. Overall, the stories illustrate how maintaining physical activity was negotiated daily in terms of participants’ time, energy, and other priorities. The struggle to allocate time for physical exercise was illustrated in a photo of 2 towels (Figure 6). The participant explained that an exercise session, including showering, decreased the time available for other activities she had to do. A lack of personal strategies to navigate through these difficulties, including health challenges, was a barrier to maintaining physical activity.

#### Strategies for Integrating Physical Activity Into Daily Life

In contrast to the stories about participants’ challenges with maintaining physical activity, participants shared strategies developed to support their regular engagement in physical activity after the intervention. The goals established at the end of the intervention provided each participant with an individualized action plan for ongoing exercise and physical activity. To stay aligned with these goals, one participant shared a visual strategy of integrating them into her daily routine: “I’ve got them here all the time. Like…I’ve written them down, so I have them…together with Borg, I have them on my desk there. All the time. So I look at them [when she passes by]” (female participant, 75 years old, stroke). The attitude expressed in the quote reflects a belief in her capability to use the tools provided by the intervention and daily visual cues, both of which facilitated maintaining physical activity after the intervention. Another participant shared a sense of control over maintaining physical activity after a short-term illness: “It’s like always, when you get going with something like this, you get sick or something. But I feel, it’s fine, you know. I just continue from where I was*”* (female participant, 56 years old, stroke). In cases where participants struggled with long-term physical impairments and fatigue after stroke or TIA, a similar capability to develop strategies to overcome challenges was identified, including taking medication before exercise, balancing activity and rest, or prioritizing activities in daily life, despite their fatigue.

Although some strategies were developed by the participants themselves, other strategies had been initiated during the intervention. One such strategy was self-monitoring through the activity diary in the STAAR app. However, participants saw reporting in the app as an extra task with little added value compared with other self-monitoring methods, and it was therefore not considered a facilitator for maintaining physical activity. Instead, facilitators were tools such as handwritten exercise logs or step counters on their phones. These easily accessible tools provided both reinforcement and validation of what had been achieved while also acting as a motivational prompt, indicating the need to engage in more physical activity in order to reach the week’s goal. Similarly, the prerecorded exercise videos in the STAAR app were used only to a limited extent, as they did not meet the participants’ needs for intensity and variation. Consequently, other digital resources were explored, such as live exercise sessions broadcasted by the public service morning show. Both the activity diary for self-monitoring and the exercise videos in the STAAR app shared a common challenge: They were underused, and participants developed alternative strategies that supported them to maintain physical activity after the intervention. Among these strategies, participants emphasized the importance of planning and routine, such as following a preplanned weekly schedule or exercising in the mornings to ensure that physical activity happened. One participant shared adhering to a structured yet feasible action plan:

*So, if I’ve decided on Tuesday and Thursday, and Tuesday turns out crap, then I just do Wednesday instead. It’s not the end of the world. I mean…just switch the day. You don’t have to be that rigid. Just because it didn’t work on Tuesday doesn’t mean I have to wait until next Tuesday. I mean…I think that’s exactly why you have these planned routines—so it’s actually feasible*.

[Female participant, 56 years old, stroke]

As illustrated by the quote, adapting the action plan for exercise enabled the participants to overcome barriers like fatigue or difficulties balancing physical activities with other engagement, roles, and responsibilities in daily life.

### The Meaning of Context for Maintaining Physical Activity After the Intervention

The participants’ context—including dynamic interactions with their social and physical environments—was crucial for keeping the physical activity bubble afloat after the intervention. Within their immediate context, they identified significant others who could ease the transition from the physical therapist’s supportive role, as well as meaningful places that offered favorable conditions to maintain physical activity.

Being part of the intervention entailed continuous social support for physical activity over 6 months. At completion, the participants missed both the guidance from the physical therapist and the social context provided by the regular exercise sessions. However, the participants identified alternative resources within their social context that facilitated the maintenance of physical activity after the intervention. One participant shared how taking part in the intervention made him realize the importance of having a companion to make it to the gym and identified a key person within his social network. He said:

My wife and I go to the gym two or three times a week, and it’s important that she’s with me. Otherwise, I might not go. She’s great! I was thinking maybe I needed a personal trainer, but I don’t. I just need someone to go with. And now I have that.

[Male participant, 56 years old, stroke]

In this way, having scheduled sessions with a companion fostered a sense of accountability, as someone was expecting the participant to attend. In addition, engaging in physical exercise with a companion provided a person who checked in on progress, contributed to a sense of safety, and gave feedback—similar to the support offered by the physical therapist during the intervention. Exercising with another person also brought joy and added value beyond physical activity itself. One participant described how the exercise class helped her build relationships and make friends, which facilitated her continued participation. Moreover, encouragement from significant others, such as friends, neighbors, and family members, facilitated maintaining physical activity by reinforcing the participants’ decision to stay active and strengthening their sense of doing something positive for themselves. One participant illustrated this motivational support by quoting how her children encouraged her to prioritize engagement in physical exercise:

*“’No, don’t skip your physical exercise, we can meet up later’ or ‘No, do that first, it’s what matters most to you’ and stuff like that. They always cheer me on. They get that this has been really good for me, and they see that I’ve become a…I think I’ve changed quite a bit. Not in huge ways, but yeah, they can see that physical exercise really does me good*.

[Female participant, 67 years old, TIA]

**Figure 7.**
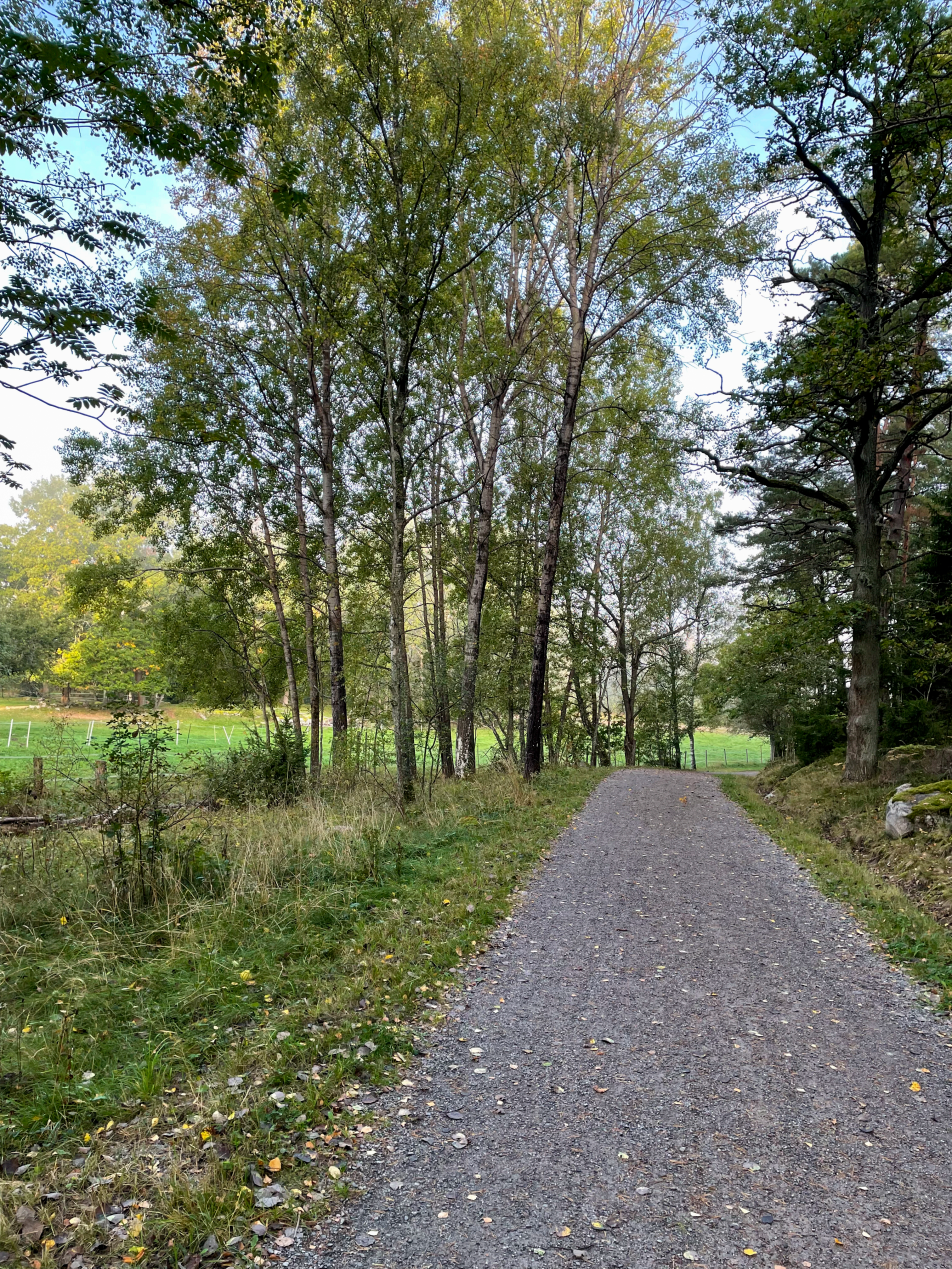
A gravel trail illustrating a designated place facilitating a meaningful physical activity experience (female participant, 75 years old, stroke).

**Figure 8.**
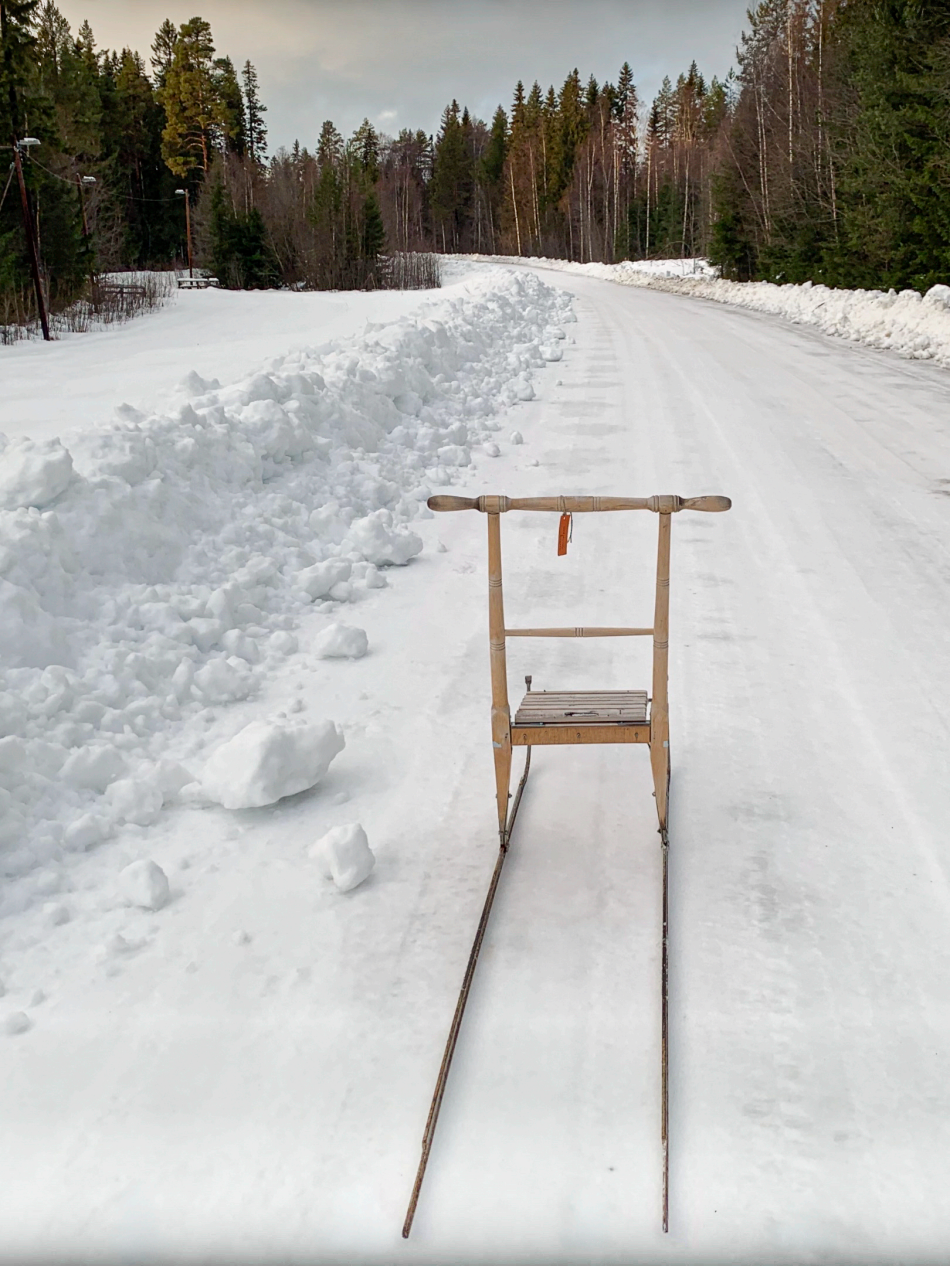
A kicksled illustrating an adaptation to maintain physical activity during the winter season (female participant, 76 years old, stroke).

Alongside the social context, having a designated place to engage in physical activity served as a valuable resource that facilitated maintenance following the intervention. One factor for this place was accessibility, including geographical proximity and ease of access via transportation, contributing to a lowered threshold for exercising, as illustrated by one participant: “I like it when it’s nearby and easy to get to and all that. That’s kind of my problem—when it gets complicated or takes longer than I had in mind, I lose motivation” (female participant, 67 years old, TIA). The outdoor environment was one such example. In addition, engaging in physical activity outdoors provided mental rest through its calm and quiet atmosphere—something particularly valued by participants experiencing fatigue. It could also provide a sense of familiarity; one participant described feelings of comfort in her outdoor walking routine, as she was well-acquainted with the route’s length and the time it typically took to complete. In one photo of a gravel trail (Figure 7), a participant captured how outside places could offer a meaningful nature experience and elaborated on how seasonal changes and the beautiful wildlife inspired her to take daily walks. However, using outdoor environments for daily physical activity was also associated with barriers related to seasonal and weather changes, especially rainy days or when the roads were slippery in the winter. For the participants, these barriers led to disruptions in their exercise routines—sometimes resulting in doing less than planned and, at other times, failing to adhere to the goals they had set at the end of the intervention. To overcome the weather and seasonal challenges, the participants needed to adapt their physical activity behaviors. One participant shared a photo of a kicksled (Figure 8) that exemplified this adapted behavior and how a kicksled enabled outdoor physical activity during the winter. For others, a pair of stable walking shoes or walking sticks helped maintain physical activity, through providing safety and inspiring them to go out for a walk regardless of the weather.

## Discussion

### Principal Findings

This study provides insights into barriers and facilitators of maintaining physical activity after completing an mHealth intervention from the perspective of individuals poststroke or TIA. The findings show how maintaining physical activity following an mHealth intervention is founded on a reciprocal relationship among the personal factors, behavior, and the environment. These findings align with behavioral change theories such as SCT [19], and the discussion will focus on aspects of personal, environmental, and behavioral factors for maintaining physical activity after an mHealth intervention.

This study found that personal factors, including positive experiences with regular physical exercise and knowledge acquired through interaction with a physical therapist, supported awareness of the health benefits of physical activity and habit formation. Consequently, these factors helped individuals incorporate physical activity into their daily routines and adjust their behaviors to follow physical activity guidelines. Similar findings were reported by Wahlich et al [36] who found that inactive mid-life and older adults made lifestyle changes in their daily lives and increased their awareness of the importance of physical activity after a pedometer-based walking intervention. Although confirming the findings of Wahlich et al [36], our study also demonstrated that participants not only acquired knowledge but also actively applied it by developing and adapting individual exercise routines after the intervention. This process was supported during the intervention by the creation of a social environment, which included support provided by the physical therapist who had extensive experience in stroke rehabilitation and played a key role by providing knowledge, reinforcement, and encouragement to maintain being physically active. After the intervention, this external support provided by the social environment was succeeded by participants’ own experiences with positive outcomes, such as reduced pain and improved well-being, which, in line with SCT [37], further reinforced them to maintain regular physical activity.

The discontinuation of support from the physical therapist was perceived as a barrier to continued engagement in physical activity after the intervention. Supervision is recognized as an important component to supporting behavior change in individuals poststroke [38] but is inherently time-limited and resource-intensive. These constraints highlights the importance of identifying and strengthening alternative support once structured supervision ends. Our findings show how participants identified resources within their social network to substitute the physical therapist’s supportive role during the intervention. These results are in line with previous findings highlighting that encouragement from significant others (eg, neighbors, friends, and family), as well as engaging in physical activity with a companion can facilitate maintaining physical activity [17,18,36]. The transition of support to the individual’s own social network suggests, consistent with SCT [39], that individuals poststroke or TIA can sustain behavior change by developing alternative sources of social support within their immediate context following an mHealth intervention. This is an important finding, highlighting the need to incorporate strategies and training into mHealth interventions that help individuals integrate physical activity into daily life, supported by their social and environmental contexts.

Participants developed and used various personal strategies, some introduced during the intervention, to facilitate maintenance of physical activity. One such strategy was the self-monitoring of physical activity, where participants tracked their activity levels. The reported preference for using step counters on their mobile phone to monitor their physical activity was consistent with findings on strategies that facilitated physical activity maintenance in adult and older populations [17,18]. Handwritten exercise logs were preferably used, while the self-monitoring tool within the app was used to a lesser extent, possibly because it did not fully meet the needs of people poststroke or TIA. Our findings align with a previous quantitative evaluation of the mHealth version of the i-REBOUND intervention, which showed a decline in engagement with the app’s self-managed tools over time [40]. This decline may reflect limited integration of key features known to enhance app engagement, such as personalization, adaptive content, and continuous feedback [41]. Although this study provides new insights into personal and environmental factors influencing maintenance—particularly those not directly related to the digital tools—further research is needed to better support long-term engagement in physical activity through mHealth solutions after stroke or TIA. Future refinements of the app may benefit from the development of more personalized content with regular updates and from the integration of automated self-monitoring of daily step counts or minutes in moderate physical activity, thereby improving its usability and relevance for this population. In SCT, self-monitoring is an important self-regulatory strategy that enables individuals to track their behavior, evaluate progress, and make necessary adjustments to achieve their goals [42]. In addition, SCT also emphasizes that maintaining physical activity requires the development of other self-regulatory capabilities, such as goal-setting and planning, as well to help individuals navigate barriers and maintain motivation [39]. These self-regulatory strategies were reflected in this study, demonstrating how participants applied the knowledge acquired from the intervention, along with their prior experience, and developed strategies for overcoming barriers. However, this study also showed that participants with limited prior knowledge of physical exercise and limited personal resources to develop strategies face greater challenges in implementing strategies for maintaining physical activity.

This study demonstrated that health-related challenges, including comorbidities, short-term illnesses, and stroke-related symptoms, were substantial barriers to maintaining physical activity after the mHealth intervention. The impact of comorbidities is an especially important consideration for health promotion interventions targeting individuals poststroke or TIA, as comorbidities are more prevalent in this group than in the general population and have a substantial negative influence on both functional outcomes and mortality [43,44]. In addition, comorbidity is strongly linked to age, with most adults older than 65 years having one or more diagnoses, and because people poststroke or TIA are often older, age-related comorbidities are frequent [45]. Furthermore, consistent with prior research [36,46,47], the participants struggled to allocate time for physical activity along with other responsibilities and engagements. These findings underscore that maintaining physical activity could be challenging for people poststroke or TIA given the outlined health-related and contextual barriers. Hence, these circumstances should be carefully considered in the design and development of mHealth interventions. Building on this study’s findings that personal strategies can help overcome health-related and contextual barriers, mHealth interventions should include components that support the implementation of such strategies in complex life situations. These components should balance the reinforcement and adaptation of existing strategies among experienced and resourceful participants, with targeted support for those who face greater challenges in the implementation.

### Implications

Our results highlight the important consideration of the interaction between individuals and their environment while supporting the transition from therapeutic support to self-management in mHealth physical activity interventions for people poststroke or TIA. One potential approach is to include a structured maintenance phase in the intervention design, during which therapeutic support is gradually reduced. In this phase, participants can progressively take more responsibility for their own development and independently apply self-regulation techniques to sustain physical activity. Because behavior maintenance is a dynamic process, discontinuities are to be expected; therefore, training participants to manage setbacks is recommended [39], including strategies that foster agency and resilience to overcome health-related and contextual barriers. Our findings further indicate that support from significant others, through encouragement and companionship, is essential for maintaining physical activity. Accordingly, future mHealth interventions should be aware of the complex situation for this population and help participants identify personal strategies in daily life and engage with a supportive social environment beyond the formal intervention setting. Finally, the study findings should be interpreted within the Nordic context, as, for instance, different climates, seasonal contexts, or digital health care systems may yield distinct barriers and facilitators.

### Strengths and Limitations

A major strength for the study’s trustworthiness was the design and methodology, which contributed to the rich dataset developed through repeated semistructured interviews at 3 months and 6 months postintervention and participant-generated photographs during the second interview. This approach facilitates going beyond surface-level descriptions of participants’ experiences by building rapport and stimulating reflection between and during sessions [23,34,48]. Further, using participant-generated photos may help reduce power imbalances between researcher and participant while also enabling the introduction of new dimensions to data generation that might not be anticipated in the interview guide, thereby contributing to a deeper understanding of the issue under study [23,34]. Furthermore, participant-generated photos and interviews at 3 months and 6 months contributed to reducing recall and response bias and to gaining both short- and long-term experience with maintaining physical activity. Additional follow-up could have provided more insight into maintaining physical activity. The design was grounded in a constructivist epistemology, with reflexivity as a central component in the generation and analysis of data [26,27]. The first author had brief contact with the participants during the feasibility trial’s data collection phase. This dual role may induce the risk of social desirability bias. In addition to the design with repeated interviews and participant-generated photos, several strategies were used to elicit authentic interview responses, such as providing assurances and probing questions [49]. These strategies for an authentic and rich dataset were developed through reflexive discussions of pilot interviews, followed by continuous reflexivity through data generation.

Limitations of the study include a lack of diversity in some of the aspects of the sample recruited from the mHealth version of the i-REBOUND intervention [21]. Diversity was obtained in terms of geography, age, educational background, and experiences with daily use of the internet and mobile apps. However, the relatively high physical activity and functional level, gender imbalance, and lack of a more complete socioeconomic background represent limitations that influence the transferability of our findings. The challenges with recruiting a diverse sample were previously addressed [21,50]. Although the participants in this study were a relatively homogeneous group, they still faced a variety of barriers related to maintaining physical activity after the mHealth intervention and needed support to adjust their physical activity to meet recommendations given their current life situations. However, our findings show important facilitators, such as knowledge acquisition and support from the social and environmental context, that can be useful for exploring conditions for more diverse populations. To facilitate the judgment of transferability to other contexts and populations, we reported detailed descriptions of the study’s methodology [27,51]. To explore how gendered and socioeconomic aspects as well as experiences of those with a more sedentary lifestyle and severe stroke influence physical activity maintenance, recruitment strategies in future studies should aim for a more diverse sample, not the least because socioeconomic disparities are shown to be related to stroke incidence and outcome [52].

### Conclusions

This study points out the need for future mHealth interventions to better prepare people poststroke and TIA to develop and apply personalized strategies for managing barriers to maintaining physical activity. The complex life situation following stroke or TIA, involving health-related challenges and contextual barriers, can limit individuals’ ability to engage in regular physical activity. Nevertheless, participants may develop strategies, supported by knowledge and tools from mHealth interventions and the individuals’ surrounding context, to navigate these challenges. These insights highlight opportunities for future mHealth interventions to strengthen the interaction between individuals and their environment while also empowering personalized strategies for supporting behavior change and long-term physical activity maintenance.

## Abbreviations

mHealth: mobile health
SCT: social cognitive theory
STAAR: Stroke Treatment through Active and Accessible Rehabilitation
TIA: transient ischemic attack
